# Insight into Crosstalk between Ferroptosis and Necroptosis: Novel Therapeutics in Ischemic Stroke

**DOI:** 10.1155/2021/9991001

**Published:** 2021-06-25

**Authors:** Yue Zhou, Jun Liao, Zhigang Mei, Xun Liu, Jinwen Ge

**Affiliations:** ^1^College of Integrated Traditional Chinese and Western Medicine, Hunan University of Chinese Medicine, Changsha, Hunan 410208, China; ^2^Medical School, Hunan University of Chinese Medicine, Changsha, Hunan 410208, China; ^3^Third-Grade Pharmacological Laboratory on Chinese Medicine Approved by State Administration of Traditional Chinese Medicine, Medical College of China Three Gorges University, Yichang, Hubei 443002, China; ^4^School of Medicine, Shaoyang University, Shaoyang, Hunan 422000, China

## Abstract

Ferroptosis is a nonapoptotic form of cell death characterized by iron-dependent accumulation of lipid hydroperoxides to lethal levels. Necroptosis, an alternative form of programmed necrosis, is regulated by receptor-interacting protein (RIP) 1 activation and by RIP3 and mixed-lineage kinase domain-like (MLKL) phosphorylation. Ferroptosis and necroptosis both play important roles in the pathological progress in ischemic stroke, which is a complex brain disease regulated by several cell death pathways. In the past few years, increasing evidence has suggested that the crosstalk occurs between necroptosis and ferroptosis in ischemic stroke. However, the potential links between ferroptosis and necroptosis in ischemic stroke have not been elucidated yet. Hence, in this review, we overview and analyze the mechanism underlying the crosstalk between necroptosis and ferroptosis in ischemic stroke. And we find that iron overload, one mechanism of ferroptosis, leads to mitochondrial permeability transition pore (MPTP) opening, which aggravates RIP1 phosphorylation and contributes to necroptosis. In addition, heat shock protein 90 (HSP90) induces necroptosis and ferroptosis by promoting RIP1 phosphorylation and suppressing glutathione peroxidase 4 (GPX4) activation. In this work, we try to deliver a new perspective in the exploration of novel therapeutic targets for the treatment of ischemic stroke.

## 1. Introduction

Due to its increasing incidence, stroke is now the leading cause of serious long-term disability and death [1]. Ischemic stroke accounts for 70-80% of total stroke cases worldwide, and survivors often experience sensorimotor disorders in one or more body regions [1, 2]. During ischemia, the blood supply to brain tissues is disrupted, which subsequently promotes a cascade of pathophysiological responses resulting in different types of cell death, including autophagy, apoptosis, necroptosis, and ferroptosis [3]. Ferroptosis, a recently discovered nonapoptotic form of cell death, is characterized by iron overload, glutathione (GSH) depletion, glutathione peroxidase (GPX) 4 inactivation, and lipid and amino acid metabolic imbalances. Necroptosis, an alternative form of programmed necrosis, is regulated by receptor-interacting protein (RIP) 1 activation and by RIP3 and mixed-lineage kinase domain-like (MLKL) phosphorylation via inhibition of caspase-8. Emerging studies have reported that ferroptosis and necroptosis both induce and aggravate brain tissue damage following the onset and development of cerebral ischemia and cerebral ischemia/reperfusion injury (CIRI) [4–8]. Regarding therapy, tissue plasminogen activator (t-PA), the only thrombolytic drug approved by the Food and Drug Administration for ischemic stroke treatment, dissolves blood clots by activating a proteolytic enzyme [8]. Early application of thrombolytic drugs is beneficial for the recovery and prognosis of patients with acute ischemic stroke. However, accumulating research has revealed that thrombolytic drugs have many contraindications and narrow therapeutic windows and are associated with a high risk of hemorrhagic transformation [9, 10]. In addition, delayed administration of recombinant t-PA (rt-PA) may lead to poor reperfusion [11, 12]. Therefore, clinical use of thrombolytic drugs has recently declined. For the above reasons, interventions targeting the specific types of programmed cell death have been investigated to provide new ideas for the treatment of ischemic stroke [13, 14]. Ferroptosis differs from necroptosis in morphological characteristics; developmental steps; and key regulators, inducers, and inhibitors (Table 1). However, increasing evidence has suggested that significant crosstalk occurs between ferroptosis and necroptosis following ischemic stroke [15–21]. In this review, we summarize the mechanism underlying the crosstalk between necroptosis and ferroptosis in ischemic stroke. Elucidation of this mechanism could provide a new perspective supporting advancement of ischemic stroke treatment.

## 2. Ferroptosis and Cerebral Ischemia

**Figure 1:**
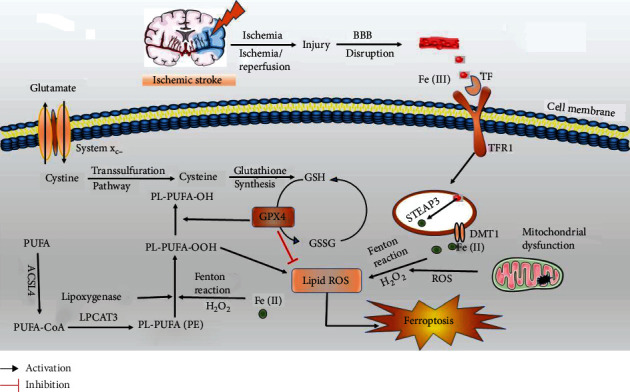
The mechanisms of ferroptosis in ischemic stroke. (1) Following ischemic stroke, BBB is disrupted, which allows Fe (III) in the blood to be released into the brain parenchyma with the cooperation of TF and TFR1. Fe (III) is transported from the endosome to the cytoplasm as Fe (II) by DMT1 with the cooperation of STEAP3. Iron overload accelerates lipid ROS accumulation and ferroptosis via Fenton reaction. (2) System x_c_- is simultaneously impaired, which inhibits cystine-glutamate exchange and decreases the generation of the antioxidant GSH and GPX4. (3) Metabolic imbalances of lipids and amino acids aggravate lipid ROS accumulation and ferroptosis. LPCAT3: lysophosphatidylcholine acyltransferase 3; H_2_O_2_: hydrogen peroxide; GSSG: oxidized glutathione.

In ischemic stroke, ferroptosis contributes to structural and functional integrity damage including blood–brain barrier (BBB) impairment, which is characterized by rapid neuronal death and dysfunction. Abundant evidence has demonstrated that the mechanisms of ferroptosis include GSH depletion [33, 34]; GPX4 inactivation [34–36]; and metabolic imbalances of iron [34, 37], lipids [36], and amino acids [38] (Figure 1). We will elucidate these mechanisms and their relationships with cerebral ischemia.

### 2.1. Iron Overload

Most iron comes from damaged or aged red blood cells; this iron is released by macrophages via transferrin receptor (TFR) 1 expressed on the cell surface [39]. Macrophages first engulf red blood cells and then use heme oxygenase to breakdown heme and eventually release iron. The excess iron in cells is stored in ferritin. A small amount of iron comes from food, and dietary iron consists of heme iron and inorganic nonheme iron [40].

Heme iron and nonheme iron are absorbed as Fe (II) by duodenal epithelial cells, and then, Fe (II) absorbed is oxidized to Fe (III) by the ferroxidase hephaestin; eventually, the Fe (III) enters the circulation via ferroportin (FPN) [41–43]. Moreover, virtually all circulating iron binds to transferrin (TF) in serum [44] to keep iron in a soluble form [42], which makes it available for absorption. TF-bound Fe (III) in circulation binds to TFR1 firstly and then Fe (III) is taken up into acidified endosomes [45]. The content of Fe (III) transported from the endosome to the cytoplasm as Fe (II) by divalent metal transporter 1 (DMT1) is reduced with the cooperation of six-transmembrane epithelial antigen of prostate 3 (STEAP3) messenger RNA (mRNA), which is expressed at high levels in macrophages and hepatocytes [46, 47]. On the other hand, overload iron in the plasma promotes non-TF-bound iron (NTBI) accumulation [41, 48].

Furthermore, iron is exported via FPN controlled by hepcidin which is the master molecule of iron negative homeostasis regulation. The expression of hepcidin is regulated by the bone morphogenetic protein (BMP)/SMAD pathway, as well as the JAK/signal transducer and activator of transcription 3 (STAT3) pathway and mitogen-activated protein kinase (MAPK)/eukaryotic protein kinase (EPK) pathway [49]. TF-bound iron causes a shift from TFR1/HFE complexes to TFR2/HFE complexes and also triggers the SMAD phosphorylation and then activates transcription gene which encodes hepcidin [49]. Interleukin- (IL-) 6 binds IL-6 receptor to initiate a JAK/STAT signaling and hepcidin expression [50]. In contrast, erythropoietin inhibits hepcidin expression and accelerates iron accumulation through the BMP/SMAD pathway and the MAPK/EPK pathway [49]. When the plasma iron concentration is high, diferric TF binds TFR2 to induce upregulation of hepcidin in hepatocytes. Besides, hepcidin binds to FPN to occlude outward open FPN and accelerate FPN degradation, which decreases iron export [51, 52].

Iron released into the circulation binds to TF and then iron is transported to sites of storage and utilization [53]. For example, iron is involved in processes such as the synthesis of some proteins, including hemoglobin and myoglobin, and redox reactions. There are multiple pathways of iron utilization by erythroblasts in mammals, including pathways involving TFR1 and the diferric TF-TFR1 complex [53]. TF and TFR are the major iron transporters under physiological conditions, instead of SLC39A14 which is a member of the solute carrier 39 family mediating cellular uptake of iron, zinc, and manganese [54, 55]. However, SLC39A14 functions as the hepatic transporter of NTBI in the absence of TF [54, 55]. Indeed, hepatocyte-specific TF-knockout (TF-LKO) mice exhibit increased serum levels of NTBI and develop iron overload in a variety of tissues [54]. Furthermore, TF-LKO mice exhibit reduced expression of TFR1 at both the mRNA and protein levels; meanwhile, TF-LKO mice exhibit increased expression of ferritin-L and ferritin-H at the protein level [54].

Normally, iron metabolism in the body is stable and beneficial. Furthermore, iron regulatory proteins (IRP) 1 and IRP2, which are central regulators of cellular iron homeostasis, are vital in the process of iron metabolism. Ferric ions bind to iron-regulatory element (IRE) with high affinity, which enables tight coordination between cellular iron uptake and ferritin/heme synthesis [56]. This tight coordination increases iron levels by repressing the translation of ferritin and maintaining the stability of TFR1 mRNA at low levels of intracellular iron [56]. As an effective redox cycling metal, iron has the potential to catalyze the production of noxious free radicals, especially in the central nervous system [57].

Ferroptosis ultimately leads to decreased neural function and/or structural integrity of the brain. In an ischemic stroke mouse model, free radical production and excessive iron have been found to lead to an oxidative stress response and neuronal death by causing prolonged upregulation of TFR1 and increasing peripheral iron uptake [58, 59]. The oxidative stress response eventually has an adverse effect on disease recovery [58, 59]. After ischemic stroke, disruption of the BBB enables excessive accumulation of intracellular or extracellular fluids in the brain, which results in brain edema and aggravates the degrees of brain tissue injury and nerve dysfunction. Numerous studies have demonstrated that BBB disruption is related to the ability of iron pools from the blood gain sudden accessing to the brain parenchyma, and overload iron aggravates ferroptosis which is induced by lipid peroxidation via Fenton's reaction [13, 60, 61]. Thus, changes in iron content in brain tissues reflect the extent of BBB dysfunction.

### 2.2. GSH Depletion and GPX4 Inactivation

Metabolism of iron plays a vital regulatory role in ferroptosis, which can be reversed by GPX4 activation and GSH production. GSH, a tripeptide containing a sulfhydryl group, is composed of glutamate, glycine, and cysteine. Besides, GSH is synthesized from cystine transported by system x_c_^−^. As a vital antioxidant, GSH plays an important role in free radical scavenging and detoxification through glutathione S-transferase and GPX4 [62, 63]. On the other hand, system x_c_^−^ impairment inhibits cystine-glutamate exchange, suppresses GSH production and GPX4 activation [64], and eventually results in ferroptosis and neuronal impairment. Indeed, the levels of GSH are decreased in stroke patients and middle cerebral artery occlusion (MCAO) animal models [65].

GPX4, a vital antioxidant enzyme, converts lipid hydroperoxides into nontoxic lipid alcohols, which prevents ferroptosis [66]. Constitutive deletion of the mouse GPX4 gene or inactivation of GPX4 adversely affects normal embryonic development [67, 68], which leads to neurological dysfunction. More importantly, inactivation of GPX4 also leads to ferroptosis when glutamine is deficient [69]. Therefore, GPX4 inactivation is a major factor in ferroptosis. Consistently, loss of GPX4 leads to ferroptosis, which manifests mainly as progressive cognitive dysfunction and impaired behavior in the context of ischemic stroke [70, 71]. Thus, inactivation or loss of the ferroptosis regulator GPX4 triggers cerebral ischemia. However, activation of the p53 tumor suppressor regulates ferroptotic responses without visibly influencing GPX4 function [72]. Furthermore, p53 positively regulates ferroptosis by inhibiting the expression of the cystine/glutamate antiporter SLC7A11 (light chain of subunit of system x_c_^−^) [73]. In addition, the expression levels of SLC7A11, GPX4, and GSH are decreased in MCAO rats [27].

### 2.3. Lipid and Amino Acid Metabolism Imbalances

The initiation and execution of ferroptosis lie at the intersection of amino acid, lipid, and iron metabolism [74]. GPX4 converts potentially toxic lipid hydroperoxide (L-OOH) to nontoxic lipid alcohol (L-OH) [74, 75]. Inactivation of GPX4 or depletion of GSH ultimately results in overwhelming lipid peroxidation that causes ferroptosis. In the central nervous system, lipids and lipid mediators are essential for maintenance of normal brain tissue structure and function. Besides, some lipids have either neuroprotective or neurodegenerative effects on poststroke brain tissue [76]. Arteriosclerosis is a major cause of stroke and associated with lipid deposition. Lentivirus-mediated A20 overexpression increases ROS generation in lipid-rich environments and enhances erastin-induced ferroptosis which is associated with GPX4 downregulation and acyl-CoA synthetase long-chain family member (ACSL) 4 upregulation [77]. Phosphorylase kinase G2 (PHKG2) regulates the availability of iron to lipoxygenase and lipids, including polyunsaturated fatty acids (PUFAs) with labile bis-allylic hydrogen atoms [78]. The abundance and localization of PUFAs determine the degrees of lipid peroxidation [78]. And free fatty acids are substrates for the synthesis of lipid signaling media, but PUFAs and free fatty acids must be esterified to membrane phospholipids and oxidized to participate in ferroptosis [78, 79]. In this process, ACSL4 catalyzes fatty acids to form acyl-CoAs which promotes fatty acid oxidation or lipid biosynthesis [80]. The research indicates that lipid oxidation upon GPX4 inhibition requires ACSL4 [80]. Further, GPX4-ACSL4 double-knockout cells show marked resistance to ferroptosis, which means ACSL4 is a component essential for ferroptosis execution and sensitive to ferroptosis [80]. Meanwhile, ACSL4 deficiency is accompanied by a significant and preferential decrease of phosphatidylethanolamine (PE) species [80]. PEs containing arachidonic acid, which are located upstream of lipid peroxidation, are key phospholipids that drive ferroptosis via oxidation and lipoxygenase. Lipid peroxidation is the driving force of cell death in ferroptosis [79], and heme-mediated lipid peroxidation may be particularly important. Heme degradation products, such as iron, have been shown to regulate inflammation, apoptosis, and antioxidant defense by heme oxygenase and isozymes, including hmox1 and hmox2 [81]. hmox1 affects vascular tension through its antioxidant activity, but hmox2 enhances cerebral blood flow during hypoxia by regulating the hydrogen sulfide pathway [81]. Furthermore, lipid peroxidation products are used as potential biomarkers of ischemic stroke. Indeed, multiple clinical studies have demonstrated that lipid peroxidation is positively correlated with the severity of neurological deficits [82, 83].

Amino acid metabolism imbalance promotes ferroptosis. The step in which glutaminolysis produces glutamate is catalyzed by glutaminase 1 and glutaminase 2 in ferroptosis [84]. Glutaminase 1 inhibitor suppresses ferroptosis and protects tissues from ischemia-reperfusion injury by ablating glutaminolysis [84]. Besides, glutaminase 2 is the p53 target gene, and upregulation of glutaminase 2 results in p53-dependent ferroptosis [85]. Glutaminolysis and the glutamine-fueled intracellular metabolic pathway [84] also contribute to cysteine deprivation and increase of glutamate levels, which activates glutamate N-methyl-D-aspartic acid receptors and accelerates neuronal iron uptake [86]. Because cysteine availability is the limiting factor for the biosynthesis of glutathione, some cells that are resistant to ferroptosis induced by system x_c_^−^ inhibitors leverage the transsulfuration pathway to biosynthesize cysteine from methionine and subsequently bypass the requirement for cystine import via the cystine/glutamate antiporter system x_c_^−^ [74]. In addition, the mevalonate pathway produces antioxidants or activates selenocysteine transfer RNA, which enhances GPX4 expression [36]. Consistent with these findings, extracellular glutamate concentrations are markedly increased in MCAO rats, which accelerates neuronal iron uptake and results in excitotoxicity-related cell death [86, 87].

## 3. Ferroptosis in CIRI

Restoring the cerebral circulation following a period of occlusion reestablishes tissue oxygenation, which leads to CIRI [88, 89]. Because reperfusion aggravates metabolic dysfunction and structural destruction, CIRI is the main factor associated with the high mortality and disability rates for ischemic stroke [90]. The extent of tissue injury is directly related to the extent of blood flow reduction and to the length of the ischemic period, and it also affects the levels of cellular adenosine triphosphate and intracellular pH [91]. Furthermore, the release of adenosine triphosphate modulates alpha 1-adrenergic receptor signaling. One study has explained that alpha 1-adrenergic receptor is critical for perfusion redistribution: activity of the receptor is a prerequisite for redistribution of cerebral blood flow, but the receptor subtype may determine the magnitude of redistribution responses [92]. Therefore, activation of the alpha 1-adrenergic receptor pathway is a potential strategy for decreasing infarct size in CIRI. On the other hand, CIRI promotes the activation of cell death programs, including apoptosis, autophagy-associated cell death, ferroptosis, and necroptosis, by pattern-recognition molecules such as toll-like receptors, which recruit and activate immune system and complement system components [93]. Related research has revealed that activation of peroxisome proliferator-activated receptor-gamma (PPAR-*γ*) suppresses toll-like receptor-mediated stimulation of dendritic cells [94], thereby inhibiting the immune system. Besides, an agonist of PPAR-*γ* reduces hematoma volume and then decreases iron content from blood accessing to the brain, which attenuates iron overload [95]. In other words, marked decreases in PPAR-*γ* expression [96] contribute to the activation of ferroptosis in CIRI. Another study has found that PPAR-*γ* and ACSL4 both promote fat deposition [97]. Besides, ACSL4 inhibition prior to reperfusion suppresses ferroptosis because low expression of ACSL4 improves GPX4 expression and reduces ferroptotic marker levels [98]. Accumulating evidence has demonstrated that ferroptosis is dependent on iron or iron-dependent ROS [99, 100]. Iron overload causes prolonged upregulation of transport receptors, increases peripheral iron uptake via the BBB, and exacerbates the risk of hemorrhagic transformation [101]. Besides, iron overload also enhances basal serum lipid peroxidation after early t-PA administration [101]. Related research has found that t-PA restores blood flow to the brain but prolonged reperfusion also results in CIRI [102]. Notably, targeted iron-mediated oxidative stress extends the time window for the treatment of ischemia or reperfusion events [58]. Lipid and amino acid metabolism is also imbalanced in CIRI. For example, the activity levels of malondialdehyde (MDA) and nitric oxide (NO) are increased, and the levels of superoxide dismutase (SOD) and GPX4 are decreased in a CIRI mouse model and an oxygen-glucose deprivation/reoxygenation (OGD/R) cell model [103]. Therefore, therapeutics for iron-mediated oxidative stress are effective for CIRI.

Mitochondria are essential for maintaining cellular homeostasis and function, and mitochondrial dysfunction plays an important role in the pathogenesis of cardiovascular and neurodegenerative diseases [104]. Mitochondria-targeted antioxidant Mito-TEMPO obviously rescues doxorubicin cardiomyopathy, supporting oxidative damage of mitochondria as a major mechanism in ferroptosis-induced heart damage [105]. More importantly, ferrostatin-1 and iron chelation also alleviate heart failure induced by I/R in mice [105]. Another research has indicated that ubiquitin-specific protease 22, a member of the deubiquitinase family, protects against myocardial ischemia-reperfusion injury via the SIRT1-p53/SLC7A11-dependent inhibition of ferroptosis-induced cardiomyocyte death [106]. These findings highlight that targeting ferroptosis serves as a cardioprotective strategy for cardiomyopathy prevention [107, 108]. The similar research in the central nervous system indicated that ferroptosis may be also an emerging target in CIRI [109]. A study has found that the levels of lncRNA PVT1 are upregulated and miR-214 levels are downregulated in plasma of acute ischemic stroke patients [109]. PVT1 silencing or miR-214 overexpression significantly reduces infarct size and suppresses ferroptosis in CIRI mice. PVT1 overexpression or miR-214 silencing markedly abolishes the effects of ferrostatin-1 on ferroptosis indicators except for TFR1 expression [109]. Carthamin yellow, a flavonoid compound extracted from safflower, has been reported to inhibit Fe (II) and ROS accumulation and reverse ACSL4, TFR1, GPX4, and ferritin heavy chain 1 protein expression levels in the brain of CIRI rats [110].

As the protein associated with iron metabolism, TFR has two types, TFR1 and TFR2. The expression of TFR1 is increased in the cerebral cortex and hippocampus on the ischemic side [111]. TFR2 is an iron modulator transcribed in two isoforms, TFR2*α* and TFR2*β* [112]. It has been reported that TFR2*β* increased in wild-type mouse hearts subject to I/R, and both TFR2*β* null mouse hearts are protected against I/R injury (about 40% smaller infarct size compared to wild-type mouse hearts) [112]. TFR2*β*-KI (lacking TFR2*β* mouse model) hearts have showed an increased ferritin heavy chain and a decreased FPN1, while LCKO-KI (selective inactivation of liver TFR2*α* in KI mice) hearts have presented an upregulation of ferritin-L chain and DMT1/hepcidin-RNA [112]. Another central nerve study indicated that TFR2*β* deletion exerts neuroprotection against dopaminergic degeneration and against Parkinson's disease- and aging-related iron overload [113]. However, the efficacy of the regulation of TFR2*β* on the CIRI remains unclear; further research should be carried out.

## 4. Necroptosis

Cells undergoing necroptosis have the morphological characteristics of necrotic cells and signal regulation similar to that of apoptotic cells. Necroptosis is a programmed cell death pathway under the precise regulation of a series of intracellular factors [88, 114]. The main morphological manifestations of necroptosis are membrane pore formation, cell swelling, cell membrane rupture, and cell content release [7]. In addition, necroptosis is mediated by RIP1 activation and RIP3 and MLKL phosphorylation [115, 116].

**Figure 2:**
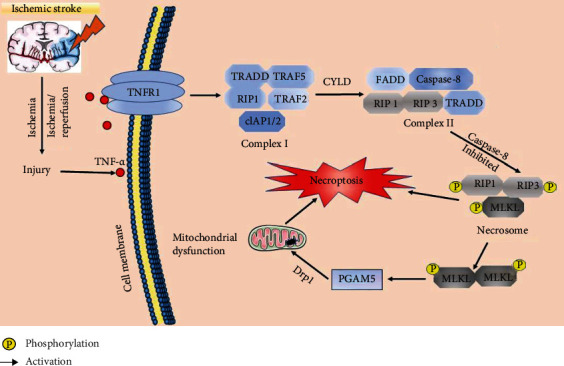
The mechanisms of necroptosis in ischemic stroke. After ischemic stroke, the content of TNF-*α* is increased and then it triggers the formation of complex I and complex II which converts into necrosome in the presence of caspase-8 inhibition. In this process, the change of RIP1 activation and RIP3 and MLKL phosphorylation accelerate necroptosis. TRADD: TNF receptor-associated death domain; TRAF: TNF receptor-associated factor; FADD: Fas-associating with death domain; cIAP: cellular inhibitors of apoptosis protein.

Coordinated and interdependent RIP1 phosphorylation and ubiquitination in the necrotic complex are important factors in necroptosis [117]. Almost half of the amino acid sequences of RIP1 and RIP3 are shared, and the topological features of these proteins are similar [118]. The intermediate domain of RIP1 contains a receptor-interacting protein homotypic interaction motif (RHIM) that binds to the RHIM in RIP3, which forms a necrosome [114]. Moreover, the critical necrosome constituents RIP1 and RIP3 play roles as signaling intermediates during MLKL activation. MLKL protein inhibition or inactivation is necessary for necroptosis [119, 120]. Necroptosis represents the intersection of apoptosis and necrosis. However, the downstream signaling pathway of necroptosis, unlike that of apoptosis, is not linked with caspase [88, 121]. Factors related to necroptosis include the tumor necrosis factor receptor (TNFR) superfamily, hypoxia, and other environmental stimuli. The TNFR superfamily, the main factor of necroptosis, has been deeply researched. Its relationship with necroptosis is shown in Figure 2.

The binding of tumor necrosis factor-alpha (TNF-*α*) and death receptors, such as TNFR1, contributes to the binding of RIP1 and death receptors [122]. Complex I, which consists of RIP1 and many death receptors, forms complex II through the deubiquitinase cylindromatosis (CYLD). When RIP1 deubiquitination and caspase-8 are inhibited, RIP3 binds to RIP1, which forms complex IIb (necrosome) including RIP1, RIP3, and MLKL. The formation of necrosome is based on the similar N-terminal kinase domain and the shared RHIM of the C-terminus between RIP1 and RIP3 [100, 123]. Necroptosis is linked with RIP1 deubiquitination. In contrast, RIP1 ubiquitination leads to the recruitment of the I kappa B kinase complex and transforming growth factor-beta-activated kinase 1, which activates the nuclear factor-kappa B (NF-*κ*B) and MAPK pathways [124–127] and subsequently suppresses programmed cell death. Furthermore, increased RIP1 ubiquitination impairs RIP1 and RIP3 phosphorylation [128]. The occurrence of necroptosis is related to MLKL phosphorylation by RIP3 [129]. In addition, the downstream factor of RIP3, calcium/calmodulin-dependent protein kinase II, increases ROS levels to induce mitochondrial dysfunction [130]. Phosphorylation of RIP3 and MLKL activates phosphoglycerate mutase family member 5 (PGAM5) and then induces necroptosis [131, 132] in collaboration with dynamin-related protein 1 (Drp1) which is a dynamics-related protein that mediates mitochondrial fission, fusion, and mitophagy [133]. Moreover, RIP3 induces mitochondrial permeability transition pore (MPTP) opening via the endoplasmic reticulum stress/calcium overload/ROS pathway [114]. Therefore, the RIP1/RIP3 complex and MLKL phosphorylation are key participants in and specific biochemical markers of necroptosis [134].

## 5. Necroptosis in Cerebral Ischemia and CIRI

Necroptosis has two different outcomes for disease progression. On the one hand, necroptosis promotes cell death and neuroinflammation in the contexts of several neurodegenerative conditions [135] and then induces cardiomyocyte injury [136]. On the other hand, necroptosis may produce an immune response, which prevents tumor progression or produces an immunosuppressive microenvironment [137]. However, the recruited inflammatory response promotes tumor progression [137]. Specifically, necroptosis attenuates inflammation induced by TNF-*α* and lipopolysaccharide, which is beneficial to intracellular pathogens that trigger this type of cell death by dampening the host immune response [138].

Sequential expression of TNF-*α* is found primarily in the neurons and glia of the infarction core in ischemic stroke, and dying cells are also detected in this area [139]. These above indicate that stimulation of the Fas/TNFR family triggers cell death and then aggravates cerebral ischemia and CIRI [6, 129]. Relevant research has proven that necroptosis activation leads to acute injury in the infarct area of an MCAO/reperfusion (MCAO/R) mouse model [8]. Besides, changes of necroptosis markers are time dependent in CIRI, and the peak time of necroptosis is 12 hours after reperfusion [140]. The findings indicate that RIP3 deletion, MLKL deletion, or necroptosis loss-of-function is the potential therapeutic strategy for neuroprotection in ischemic stroke [8].

Microglia, the primary immune cells of the central nervous system, undergo necroptosis in diverse pathological processes. Microglia are macrophage-like cells of the central nervous system with two possible phenotypes: the M1 phenotype, which expresses proinflammatory factors, and the M2 phenotype, which expresses anti-inflammatory factors [141]. Activated microglia release proinflammatory cytokines and promote cell necroptosis [70]. In one study, a model simulating ischemia is constructed with neurons expressing RIP3, and these neurons produce proinflammatory cytokines such as IL-18 and TNF-*α* in vitro [142]. In contrast, ischemic RIP3-deficient neurons secrete the anti-inflammatory cytokines IL-4 and IL-10 [142]. Thus, RIP3 and MLKL induce microglial polarization towards the M1 phenotype. Further research has suggested that M1 microglia and their receptors induce epithelial cell injury and BBB destruction [7].

## 6. Crosstalk between Ferroptosis and Necroptosis

**Figure 3:**
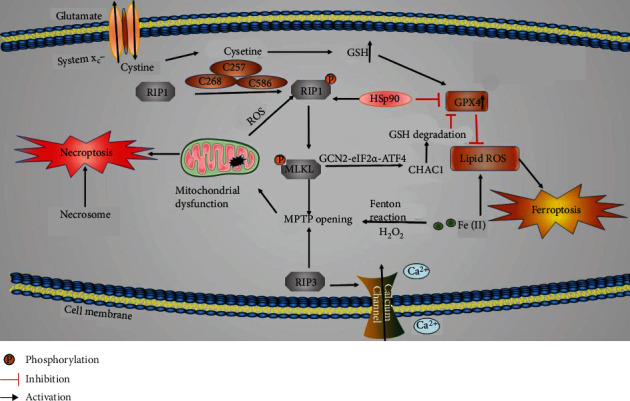
The mechanisms of signaling crosstalk between ferroptosis and necroptosis in ischemic stroke. Cysteine, HSP90, and MPTP opening are the positive factors of necroptosis; HSP90 and MPTP opening accelerate ferroptosis, while cysteine suppresses ferroptosis by promoting GSH formation.

Ferroptosis and necroptosis are different forms of cell death, but multiple lines of structural, functional, and mechanistic evidence indicate that crosstalk occurs between them (Figure 3).

### 6.1. MPTP Opening

Ferroptosis is characterized morphologically as follows: the presence of abnormally small normal mitochondria with condensed mitochondrial membranes; decreased numbers of, or a lack of, mitochondrial cristae; outer mitochondrial membrane rupture; and an electron lucent nucleus [73, 143, 144]. Moreover, nuclear membrane damage is induced prior to cytoplasmic membrane damage in ferroptosis [144]. Necroptosis is morphologically characterized by cellular organelle swelling, cell membrane rupture, and dilation of the perinuclear space [7, 144]. These results show that the structural changes associated with ferroptosis occur mainly in mitochondria and are characterized by mitochondrial atrophy, while the structural changes associated with necroptosis occur in multiple organelles, including mitochondria [145, 146]. However, these changes eventually result in cell membrane rupture, mitochondrial membrane potential depolarization, and MPTP opening. MPTP opening leads to mitochondrial energetic dysfunction, organelle swelling, rupture [147], and typically ferroptosis [148] and necroptosis [149]. Ischemic stroke and subsequent CIRI promote ROS production in the mitochondria of neuronal cells, and the MPTP is deeply involved in this process [150]. Some studies have shown that RIP3 upregulation leads to calcium influx, calcium/calmodulin-dependent protein kinase II activation, xanthine oxidase expression, and excess ROS production in the ischemic environment [149]. Meanwhile, RIP3 upregulation also induces MPTP opening via endoplasmic reticulum-calcium-xanthine oxidase signaling pathways [149, 151–154]. The changes above eventually result in increased membrane permeability and mitochondrial swelling and dysfunction [149, 151–154]. In addition, necrosome promotes MPTP opening and ROS generation, which ultimately leads to TNF-*α*-independent necroptosis [20]. GSH reduction and GPX4 inhibition contribute to lipoxygenase activation and calcium influx, which induces MPTP opening and mitochondrial dysfunction [18]. On the other hand, lipoxygenase activation and GPX4 inhibition aggravate lipid peroxide production, which induces ferroptosis formation [155]. Iron overload, one of the mechanisms of ferroptosis, has been shown to trigger MPTP opening and necroptosis via induction of ROS accumulation in osteoblastic cells [156]. Furthermore, ROS-mediated endoplasmic reticulum stress and mitochondrial dysfunction are known to promote structural and functional injury in the nervous system [157]. Other studies have indicated that endoplasmic reticulum stress is linked with ferroptosis via toxic lipid peroxides [158]. Besides, ferroptosis and endoplasmic reticulum stress response activation are induced by system x_c_^−^ inhibition [158]. Aside from cell membrane and mitochondrial injury, another common pathological feature of ferroptosis and necroptosis is BBB damage. Notably, macrophage and microglial activation is found in ferroptotic tissue [17].

### 6.2. Cysteine and HSP90

The cysteine plays important roles in both ferroptosis and necroptosis in the central nervous system. Three cysteines (C257, C268 and C586) in RIP1 form intermolecular disulfide bonds, which induce ROS production, subsequently inducing RIP1 autophosphorylation on serine residue 161. The autophosphorylation enables RIP1 to recruit RIP3, which forms a functional necrosome [159]. In addition, cysteine can be converted to cystine in most tissues. As a cystine/glutamate antiporter, SLC7A11 transports cystine to downregulate ferroptosis [38]. Therefore, SLC7A11 plays a key role in antioxidant defense. Besides a key regulator of ferroptosis, SLC7A11 is also a target for coordinating immunotherapy with radiotherapy [160]. For example, SLC7A11 inhibition leads to mixed-type cell death via ferroptosis and necroptosis in a context-dependent manner in hepatocellular carcinoma cells [161].

Moreover, some studies have found that substances containing cysteine play vital roles in the outcomes of ferroptosis and necroptosis. For example, one novel study found that knockdown of progranulin which is a secreted glycoprotein and cysteine-rich growth factor, significantly promotes necroptosis in MCAO mice [162]. In addition, progranulin-regulated ischemic stroke is associated with ROS accumulation [162]. MCAO mice with progranulin knockdown manifest severe oxidative stress, as evidenced by increased MDA content and reduced SOD activity [162]. Therefore, progranulin is a common regulator in ferroptosis and necroptosis. Heat shock protein 90 (HSP90) contains 6 cysteines, and the expression of HSP90 is increased during OGD injury [19]. HSP90 not only has beneficial influences in cells but also stabilizes some death signal proteins and promotes cell death [19, 163]. In addition, Triad3A, an E3 ubiquitin-protein ligase, promotes the downregulation of RIP1 [21]. RIP1 forms a complex with Triad3A and HSP90. TNF-*α*-initiated stimulation does not alter the binding of HSP90 to RIP1, which means that both Triad3A and HSP90 may cooperatively regulate the homeostasis of RIP1 [21]. The finding also indicates that HSP90 promotes the formation of the RIP1/RIP3 complex [19]. Another study has indicated that HSP90-associated chaperone-mediated autophagy promotes GPX4 degradation and ferroptosis formation by regulation of LAMP2A stability [16]. An inhibitor of HSP90, 2-amino-5-chloro-N,3-dimethylbenzamide (CDDO), blocks necroptosis by inhibiting RIP1 activation, which means HSP90 accelerates RIP1 phosphorylation. Furthermore, consistent with the interaction of HSP90 and GPX4 [15, 16], CDDO suppresses GPX4 degradation and ferroptosis formation by blocking chaperone-mediated autophagy [164]. Another HSP90 inhibitor tanespimycin (17-allylamino-17-demethoxygeldanamycin) has been proved to exert inhibitory effect on both necroptosis and ferroptosis in HT-22 cells treated with TNF-*α*/zVAD.fmk or erastin [164]. Tanespimycin also improves neurobehavior of subarachnoid hemorrhage rats via HSP90/RIP3/NOD-like receptor family pyrin-domain containing 3 (NLRP3) signaling pathway [165]. Except for that, general control nonderepressible 2 (GCN2) expression is increased in MCAO mice [166]. Besides, HSP90 improves the activity and lever of GCN2 [167]. Further research has proven that the GCN2-eIF2*α*-activating transcription factor 4 (ATF4) pathway is the downstream of RIP1-RIP3-MLKL. And ATF4-regulated gene, glutathione-specific gamma-glutamylcyclotransferase 1 (CHAC1), is the downstream of GCN2-eIF2*α*-ATF4 pathway [168]. Cystine starvation induces necroptosis and ferroptosis through the activated GCN2-eIF2*α*-ATF4 pathway in the triple-negative breast cancer cells [168]. Therefore, we speculate that as a common regulatory node between necroptosis and ferroptosis, HSP90 is the potential therapeutic target and its inhibitor suppresses necroptosis and ferroptosis in ischemic stroke through the GCN2-eIF2*α*-ATF4 signaling pathway.

Based on the mechanisms of ferroptosis and necroptosis, the mechanisms of drugs against ischemic stroke targeting ferroptosis or necroptosis are explained from several perspectives below (Tables 2 and 3).

## 7. Pharmacotherapies for Ischemic Stroke Targeting Ferroptosis and Necroptosis

At present, the clinical treatment of ischemic stroke involves intervention measures to restore blood flow via drug-based or mechanical thrombolysis. However, these measures have limited success, and there are no effective intervention or treatment measures to protect the brain against cell death [169, 170]. Accumulating evidence demonstrates that ferroptosis accelerates ischemic stroke, and ferroptosis inhibition can significantly reduce disease severity and facilitate functional recovery [170].

Deferoxamine (DFO), an iron chelator widely used to treat iron overload, reduces the cerebral infarct volume in MCAO rats and suppresses ferroptosis by promoting erythropoietin synthesis and increasing hypoxia-inducible factor-alpha (HIF-*α*) levels [24]. Although HIF-*α* plays an adverse role in ischemic stroke [171], it induces human umbilical cord blood hematopoietic stem cells to produce Epac1, which improves cerebral blood flow and promotes neurite outgrowth following ischemic stroke [172]. Ferroptosis is also suppressed by lipophilic antioxidants, including statins and vitamin B12. For example, as inhibitors of the hydroxymethylglutaryl-CoA reductase enzyme, statins inhibit lipid biosynthetic pathway, which reduces infarct size, neurological deficits, and adverse events in 1712 patients with acute ischemic stroke [173]. Apart from exhibiting lipid-lowering activity, statins have also been proven to be effective in improving endothelial function, increasing early reperfusion [174], and attenuating inflammatory and oxidative stress-related damage [175]. These effects reduce lipid peroxidation, protect the BBB, and prevent free iron from entering brain tissue, which suppresses ferroptosis. Homocysteine has been proven to be an adverse factor in ischemic stroke, because homocysteine activates microglia and induces proinflammatory cytokine release and promotes lipid peroxidation. Therefore, vitamin B12 (cyanocobalamin) reduces oxidative stress and lipid peroxidation by lowering homocysteine levels [25]. The changes are beneficial to inhibit ferroptosis, protect the integrity of endothelial cells and the BBB, promote neural repair, and improve recovery in ischemic stroke [25, 176, 177]. In addition to vitamin B12, the most potent compound 2-(1-(4-(4-methylpiperazin-1-yl) phenyl) ethyl)-10H-phenothiazine (51) of promethazine has a good ability to permeate the BBB and good therapeutic effect in MCAO model [178]. Meanwhile, it also suppresses erastin-induced ferroptosis [178]. Leptin is an adipocyte-derived hormone that acts as inhibiting glutamate release in the hippocampal CA3 field [179], which attenuates ferroptosis induced by glutamate excitotoxicity [180], increases cerebral blood flow in hypoperfused rat brains, protects neurologic function, and reduces infarct size [181, 182]. However, a research found that the leptin level is high in patients and mice after acute ischemic stroke [183]. The high leptin lever upregulates inflammatory factor lever [183], mediates GPX4 downregulation, and accelerates iron overload, which eventually leads to ferroptosis [184]. Therefore, leptin has become a potential target for the treatment of ischemic stroke. In addition to the synthetic drugs above, Chinese herbal medicines are critical in pharmacotherapies for ischemic stroke. For example, naotaifang, a compound Chinese herbal medicine, reduces the ROS levels and lipid peroxidation production [27]. It also increases the expression of GPX4 and GSH, which inhibits ferroptosis via the TFR1/DMT1 and SCL7A11/GPX4 pathways [27]. Apart from traditional Chinese medicinal compounds, the effects of the chemical components of Chinese herbs in pharmacotherapies for ischemic stroke have received increasing attention. Carvacrol has been found to reduce lipid peroxidation levels and increase GPX4 expression, which help suppress ferroptosis and protect the structure and function of hippocampal neurons in an ischemic stroke gerbil model [26]. Schisandrin may be a potential therapeutic agent targeting ferroptosis in ischemic stroke because schisandrin A protects mitochondria and eliminates excessive ROS [185], thereby reducing lipid peroxidation levels to inhibit ferroptosis.

In addition to pharmacotherapies targeting ferroptosis, other pharmacotherapies targeting necroptosis and the crosstalk between ferroptosis and necroptosis in ischemic stroke have also been further researched. HSP90 is the common regulator of ferroptosis and necroptosis. The selective HSP90 inhibitor17-dimethylaminoethylamino-17-demethoxygeldanamycin (17-DMAG), which is currently undergoing clinical trials for cancer treatment, effectively inhibits BBB disruption by preventing the degradation of tight junction proteins, suppressing inflammatory responses, and decreasing HSP90 expression after ischemic stroke [186]. Necroptosis inhibitors also prevent other forms of cell death, while ferroptosis inhibitors cannot. For example, the RIP1 inhibitor necrostatin-1 (Nec-1) has been proven to inhibit ferroptosis in a necroptosis/RIP1-independent manner [187]. Meanwhile, Nec-1 decreases RIP1 and RIP3 protein levels, which suppresses necroptosis and improves cognitive function [30, 188]. A potent small-molecule inhibitor of MLKL, necrosulfonamide, binds to MLKL's N-terminal CC region and reduces MLKL expression, which inhibits necroptosis, diminishes infarct volume, and improves neurological function [189]. Dabrafenib, an RIP3 inhibitor at micromolar concentrations, reduces TNF-*α* mRNA levels and attenuates TNF-*α* activation in macrophages, which decreases infarct size and protects neuron cells after focal cerebral ischemic injury [31]. As a monoclonal antibody widely used in inflammatory disease, infliximab can also be used to reduce mitochondrial damage, attenuate cytoplasmic transparency, and decrease BBB permeability and necroptosis formation in the ischemic area, eventually ameliorating neurological deficits [7]. In addition, inhibition of necroptosis-related gene expression has been intensively investigated as an ischemic stroke treatment. For example, Gsk′872 (RIP3 inhibitor) combined with RIP3 siRNA reduces the levels of RIP1, RIP3, and MLKL and MLKL phosphorylation, which protects the neurological system [171]. Chinese herbs or their active ingredients reduce the content of necroptosis-related proteins (RIP3, MLKL, and phosphorylated MLKL), which suppresses necroptosis and improves neurological function in rats following MCAO [190].

## 8. Pharmacotherapies against CIRI Targeting Ferroptosis and Necroptosis

Cerebral structural and neurological damage becomes more severe with prolonged CIRI which results from delayed diagnosis and treatment of ischemic stroke. Therefore, timely diagnosis and treatment remain critical for rapid cerebral blood flow recovery and for reducing the incidence rates of complications and recurrent stroke. However, thrombolytic drugs, which are commonly used as the therapeutics against CIRI, have declined in clinic because of their many contraindications, their narrow therapeutic windows, and the risk of hemorrhagic transformation [9, 10]. Current research seeks to explore potential therapeutic strategies for CIRI. Notably, oxidative stress, MPTP opening, cell death, and inflammation all lead to CIRI. Therefore, pharmacotherapies targeting these mechanisms are important for improving the efficacy of CIRI treatment, reducing adverse reactions, and attenuating secondary injury which results from reperfusion.

Oxidative stress plays a major role in the pathogenesis of CIRI. Some pharmacotherapies, such as metformin [191] and galangin [192], suppress ferroptosis and exert cerebroprotective effects in the context of CIRI by decreasing lipid peroxidation production or increasing the expression of GPX4. Apart from that efficacy, metformin (1,1-Dimethylbiguanide), a hypoglycemic drug, leads to glucose starvation which results in the phosphorylation and activation of adenosine 5′-monophosphate-activated protein kinase (AMPK) [193, 194]. This kinase suppresses PUFA-containing lipid biosynthesis and then inhibits ferroptosis [194, 195]. In addition to galangin, the therapeutics of other Chinese herbs and their bioactive constituents against CIRI cannot be ignored. For example, Xinshao formula, a traditional Chinese medicinal compound, exerts a protective effect against CIRI in rats by increasing the activity of SOD and GPX4 and decreasing the content of ROS and MDA [196]. Therefore, the formula attenuates oxidation or lipid peroxidation and then suppresses cell death induced by oxidative stress. The herbal carthamin yellow, as well as herb combination huangqi-honghua and its main components astragaloside IV and hydroxysafflor yellow A, significantly reduces the infarct volume after 24 h of reperfusion, increases the activity of antioxidants (e.g., SOD and GPX4), and decreases the levels of MDA, ROS, and Fe (II) [110, 197]. These botanicals may play an important role in neuroprotection by suppressing ferroptosis.

Edaravone is an upstream suppressor of ROS [150, 198]. Cyclosporine-A is a potent inhibitor of CYPD, and it acts on the prominent mediator of the MPTP [199]. The two drugs inhibit MPTP opening in ischemic stroke and then inhibit neuronal cell death [150, 198, 199]. MPTP opening is a common factor of ferroptosis and necroptosis, which is characterized primarily by RIP1 activation and RIP3 and MLKL phosphorylation. The RIP1 inhibitor Nec-1 reduces the cell death ratios of neurons after CIRI by inhibiting RIP1-RIP3 interaction and RIP3 activation [200]. Besides, the combination of Nec-1 and a glycogen synthase kinase-3 beta inhibitor can downregulate the levels of necroptosis-related proteins (RIP1, RIP3, and MLKL), decrease glial scar markers, and ameliorate chronic inflammatory responses, which suppress astrocyte necroptosis after CIRI [201]. *β*-Caryophyllene (8-methylene-4,11,11-trimethylbicyclo [7.2.0] undec-4-ene), an odoriferous bicyclic sesquiterpene, alleviates cerebral ischemic injury or CIRI by inhibiting necroptotic neuronal death and inflammatory response [202]. The therapy of emricasan (an inhibitor of caspases) combined with ponatinib (a potential inhibitor for RIP1/3) against CIRI has been reported to ameliorate necroptosis by reducing the activities of capase-8 and downregulating the expressions of RIP1, RIP3, and MLKL [203]. In addition, maresin 1, a new docosahexaenoic acid-derived proresolving agent, reduces inflammatory responses and attenuates mitochondrial damage, which may suppress TNF-*α*-induced necroptosis, diminish neuronal degeneration, and attenuate CIRI [204]. Vanillic acid (4-hydroxy-3-methoxybenzoic acid), a neuroprotective agent against CIRI, downregulates the levels of proinflammatory factors and upregulates the levels of anti-inflammatory factors [205], which can be inferred that it exerts a neuroprotective effect against TNF-*α*-induced necroptosis. Necroptosis inhibitors also attenuate ferroptosis. For example, mifepristone (11*β*-(4-dimethyl­amino)­phenyl-17*β*-hydroxy-17-(1-propynyl)­estra-4,9-dien-3-one) stimulates PPAR-*γ* to attenuate iron overload, which suppresses ferroptosis and then alleviates CIRI in rats [90]. Besides, mifepristone also inhibits inflammatory cytokines [96] to suppress necroptosis induced by TNF-*α*. As the PARP inhibitor, PJ34 (N-(6-oxo-5, 6-dihydro-phenanthridin-2-yl)-2-(N, N-dimethylamino)acetamide) enhances the DNA binding and transactivation of PPAR-*γ* [206], which inhibits PARP-related necroptosis and ferroptosis. Therefore, PJ34 promotes vascular protection and attenuates reperfusion injury induced by delayed rt-PA administration [90].

## 9. Conclusion and Perspectives

Stroke is becoming a crucial issue for people in developing countries, especially in China, where ischemic stroke has become the leading cause of death owing to its high morbidity, mortality, and disability rates. Several types of cell death pathways have been discovered. Besides, relevant research has demonstrated their roles in organismal homeostasis and the existence of crosstalk between them. Clarification of the molecular mechanisms underlying this crosstalk will not only contribute to a comprehensive understanding of the cell death machinery but also shed light on new pharmacotherapeutic targets for related diseases. Further study needs to focus on the crosstalk regarding molecular mechanisms and interplay among different types of regulated necrosis, especially ferroptosis and necroptosis. Pharmacological intervention of two or more types of regulated necrosis simultaneously may have advantages in clinic to prevent and treat ischemic stroke [207]. Nevertheless, many questions must be answered before this crosstalk can be exploited for clinical applications. For example, the roles of receptors in necroptosis/ferroptosis crosstalk have not been fully elucidated. Moreover, the precise mechanisms of some drugs, especially traditional Chinese medicine, targeting ferroptosis and necroptosis in ischemic stroke need to be further explored. In addition, thrombolytic therapy for cerebral ischemic injury has been limited due to its narrow therapeutic time window, induction of CIRI, and high risk of hemorrhagic transformation. Thus, novel therapeutic approaches, including traditional Chinese medicinal formula, that affect multiple targets and prevent neuronal death (including ferroptotic and necroptotic death) are urgently needed to be developed. Solving the problems will provide crucial support for exploiting the mechanisms of crosstalk between ferroptosis and necroptosis and intervening ischemic stroke. Overall, much work is needed before these problems can be solved.

## Figures and Tables

**Table 1:** Schematic overview of ferroptosis and necroptosis in ischemic stroke. JAK: Janus kinase; STEAP3: six-transmembrane epithelial antigen of prostate 3; ACSL4: acyl-CoA synthetase long-chain family member 4; FPN: ferroportin; TFR1: transferrin receptor 1; PHKG2: phosphorylase kinase G2; NADPH: nicotinamide adenine dinucleotide phosphate; TNFR: tumor necrosis factor receptor; DFO: deferoxamine; Nec-1: necrostatin-1.

	Ferroptosis	Necroptosis
Morphological characteristics	Shrunken mitochondria, fragmented mitochondria, enlarged cristae, dense membrane, lipid radicals	Necrosomes, ion-selective channels formed by MLKL, round and swollen cells, broken plasma membrane
Developmental steps	Iron overload, GSH depletion, GPX4 inactivation, lipid peroxidation, system x_c_^−^ impairment	RIP1 activation, RIP3 and MLKL phosphorylation
Key regulators	GPX4, JAK, STEAP3, TFR1, ACSL4, FPN, PHKG2, p53, NADPH oxidase	RIP1, RIP3, MLKL, Fas/TNFR, p53
Inducers and inhibitors	Inducers: erastin [22], sorafenib [23], acrolein [22] Inhibitors: DFO [24], vitamin B12 [25], carvacrol [26], Chinese herbal medicines including naotaifang [27]	Inducers: alkylating agents [28], X-rays [29] Inhibitors: Nec-1 [30], infliximab [7], dabrafenib [31], Chinese herbal medicines including curcumin [32]

**Table 2:** Pharmacotherapies targeting ferroptosis and necroptosis against cerebral ischemia or ischemic stroke. BCAS: bilateral common carotid artery stenosis.

Pharmacotherapy	Subject	Effects	References
*Ferroptosis*			
DFO	MCAO rats	Decreases infarct volume	[24]
Statin	Acute ischemic stroke patients	Reduces cholesterol and enhances early reperfusion	[173, 175]
Vitamin B12	Lacunar stroke patients MCAO model; one patient	Protects the BBB; improves neurological function; endothelial cell protection	[25, 176, 177]
Promethazine	HT1080 cell ferroptosis model; MCAO model	Suppresses ferroptosis; an excellent therapeutic effect; a good ability to permeate the BBB	[178]
Naotaifang	MCAO rats	Reduces ROS, MDA, and iron accumulation	[27]
Carvacrol	Ischemic stroke gerbils	Reduces lipid peroxidation levels and increases GPX4 expression	[26]
*Ferroptosis; necroptosis*			
17-DMAG	MCAO mice; OGD-subjected bEnd.3 cells	Protects the BBB; inhibits HSP90 expression; suppresses inflammation	[186]
*Necroptosis*			
Nec-1	BCAS mice	Inhibits RIP1 and RIP3 to reduce inflammation and improve cognitive function	[30]
Nec-1	MCAO rats	Decreases phosphorylated RIP1, RIP3, MLKL, and phosphorylated MLKL levels and the numbers of phosphorylated RIP1^+^ neurons	[188]
Necrosulfonamide	MCAO mice	Reduces MLKL expression and infarct volume and improves neurological function	[189]
Dabrafenib	Focal ischemic brain injury model mice	Reduces TNF-*α* mRNA levels and infarct size	[31]
Infliximab	tMCAO rats	Reduces mitochondrial damage, cytoplasm transparency, and BBB permeability	[7]
Gsk′872+RIP3 siRNA	MCAO mice; OGD-subjected HT-22 cells	Reduces RIP1, RIP3, MLKL, and phosphorylated MLKL levels to protect the neurological system	[171]
Ligustroflavone	MCAO rats	Reduces RIP3, MLKL, and phosphorylated MLKL levels to improve neurological function	[190]

**Table 3:** Pharmacotherapies against CIRI targeting ferroptosis and necroptosis. BBCAO/R: bilateral common carotid artery occlusion and reperfusion.

Pharmacotherapy	Subject	Effect	References
*Ferroptosis*			
Metformin	BBCAO/R rats	Reduces GPX, SOD, MDA, and catalase levels	[191]
Galangin	MCAO/R gerbils	Increases the expression of SLC7A11 and GPX4; hippocampal neuron protection	[192]
Xinshao formula	MCAO/R rats	Increases the activity of SOD and GPX4; decreases the activity of inducible nitric oxide synthase and the content of NO, ROS, and MDA	[196]
Carthamin yellow	MCAO/R rats	Decreases Fe (II) and ROS accumulation and MDA lever; increases GSH and GPX4 lever	[110]
Edaravone	MCAO/R rats	Reduces ROS generation, cerebral infarct size, and neurological defects	[198]
*Necroptosis*			
Cyclosporine-A	BBCAO/R rats	Inhibits MPTP opening; reduces RIP1 and RIP3 levels	[199]
Nec-1	MCAO/R rats	Suppresses RIP1-RIP3 interaction and RIP3 activation; decreases the dead rate of neurons in the hippocampal CA1 region	[200]
*β*-Caryophyllene	OGD/R neuron cell; MCAO/R mice	Decreases TNF-*α*, IL-1*β*, and toll-like receptor 4 levels; decreases RIP1 and RIP3 expression and MLKL phosphorylation	[202]
Emricasan+ponatinib	MCAO/R rats	Decreases RIP1, RIP3, and MLKL expression; reduces the activity of caspase-8	[203]

## Data Availability

The data used to support the findings of this study are available from the corresponding author upon request.

## Abbreviations

ACSL4: Acyl-CoA synthetase long-chain family member 4
AMPK: Adenosine 5′-monophosphate-activated protein kinase
ATF4: Activating transcription factor 4
BBB: Blood–brain barrier
BBCAO/R: Bilateral common carotid artery occlusion and reperfusion
BCAS: Bilateral common carotid artery stenosis
CDDO: 2-Amino-5-chloro-N,3-dimethylbenzamide
CHAC1: Glutathione-specific gamma-glutamylcyclotransferase 1
CIRI: Cerebral ischemia/reperfusion injury
DFO: Deferoxamine
DMT1: Divalent metal transporter 1
Drp1: Dynamin-related protein 1
FADD: Fas-associated protein with death domain
FPN: Ferroportin
GCN2: General control nonderepressible 2
GPX4: Glutathione peroxidase 4
GSH: Glutathione
HIF-*α*: Hypoxia-inducible factor-alpha
HSP90: Heat shock protein 90
IL: Interleukin
IRE: Iron regulatory element
IRP: Iron regulatory protein
JAK: Janus kinase
L-OOH: Lipid hydroperoxide
L-OH: Lipid alcohol
MAPK: Mitogen-activated protein kinase
MCAO: Middle cerebral artery occlusion
MCAO/R: Middle cerebral artery occlusion/reperfusion
MDA: Malondialdehyde
MLKL: Mixed-lineage kinase domain-like
MPTP: Mitochondrial permeability transition pore
mRNA: Messenger RNA
NADPH: Nicotinamide adenine dinucleotide phosphate
NF-*κ*B: Nuclear factor-kappa B
NO: Nitric oxide
NTBI: Non-transferrin-bound iron
OGD: Oxygen-glucose deprivation
OGD/R: Oxygen glucose deprivation/reoxygenation
-OH: Hydroxyl radical
PEs: Phosphatidylethanolamines
PGAM5: Phosphoglycerate mutase family member 5
PHKG2: Phosphorylase kinase G2
RHIM: Protein homotypic interaction motif
ROS: Reactive oxygen species
PPAR-*γ*: Peroxisome proliferator-activated receptor-gamma
PUFAs: Polyunsaturated fatty acids
RIP1: Receptor-interacting protein 1
rt-PA: Recombinant tissue plasminogen activator
SOD: Superoxide dismutase
STEAP3: Six-transmembrane epithelial antigen of the prostate 3
TFR1: Transferrin receptor 1
TLRs: Toll-like receptors
TNF: Tumor necrosis factor
TNFR: Tumor necrosis factor receptor
TNF-*α*: Tumor necrosis factor-alpha
t-PA: Tissue plasminogen activator
17-DMAG: 17-Dimethylaminoethylamino-17-demethoxygeldanamycin.

## References

[1] Barthels D., Das H. (2020). Current advances in ischemic stroke research and therapies. *Biochimica et Biophysica Acta (BBA) - Molecular Basis of Disease*.

[2] Hume A. W., Tasker R. A. (2020). Endothelin-1-induced ischemic damage and functional impairment is mediated primarily by NR2B-containing NMDA receptors. *Neurotoxicity Research*.

[3] Davidson S. M., Adameová A., Barile L. (2020). Mitochondrial and mitochondrial-independent pathways of myocardial cell death during ischaemia and reperfusion injury. *Journal of Cellular and Molecular Medicine*.

[4] Alim I., Caulfield J. T., Chen Y. (2019). Selenium Drives a Transcriptional Adaptive Program to Block Ferroptosis and Treat Stroke. *Cell*.

[5] She X., Lan B., Tian H., Tang B. (2020). Cross talk between ferroptosis and cerebral ischemia. *Frontiers in Neuroscience*.

[6] Degterev A., Huang Z. H., Boyce M. (2005). Chemical inhibitor of nonapoptotic cell death with therapeutic potential for ischemic brain injury. *Nature Chemical Biology*.

[7] Chen A. Q., Fang Z., Chen X. L. (2019). Microglia-derived TNF-*α* mediates endothelial necroptosis aggravating blood brain-barrier disruption after ischemic stroke. *Cell Death & Disease*.

[8] Zhang Y., Li M., Li X. (2020). Catalytically inactive RIP1 and RIP3 deficiency protect against acute ischemic stroke by inhibiting necroptosis and neuroinflammation. *Cell Death & Disease*.

[9] Bhaskar S., Stanwell P., Cordato D., Attia J., Levi C. (2018). Reperfusion therapy in acute ischemic stroke: dawn of a new era?. *BMC Neurology*.

[10] Wardlaw J. M., Murray V., Berge E. (2012). Recombinant tissue plasminogen activator for acute ischaemic stroke: an updated systematic review and meta-analysis. *The Lancet*.

[11] El Amki M., Lerouet D., Garraud M. (2018). Improved reperfusion and vasculoprotection by the poly (ADP-ribose) polymerase inhibitor PJ34 after stroke and thrombolysis in mice. *Molecular Neurobiology*.

[12] Wang W., Li M. C., Chen Q. X., Wang J. (2015). Hemorrhagic transformation after tissue plasminogen activator reperfusion therapy for ischemic stroke: mechanisms, models, and biomarkers. *Molecular Neurobiology*.

[13] Liu J., Guo Z. N., Yan X. L. (2020). Crosstalk between autophagy and ferroptosis and its putative role in ischemic stroke. *Frontiers in Cellular Neuroscience*.

[14] Liu J., Kuang F. M., Kroemer G., Klionsky D. J., Kang R., Tang D. L. (2020). Autophagy-dependent ferroptosis: machinery and regulation. *Cell Chemical Biology*.

[15] Chen C. A., Wang D. K., Yu Y. Y. (2021). Legumain promotes tubular ferroptosis by facilitating chaperone-mediated autophagy of GPX4 in AKI. *Cell Death & Disease*.

[16] Zhou B. R., Liu J., Kang R., Klionsky D. J., Kroemer G., Tang D. L. (2020). Ferroptosis is a type of autophagy-dependent cell death. *Seminars in Cancer Biology*.

[17] Proneth B., Conrad M. (2019). Ferroptosis and necroinflammation, a yet poorly explored link. *Cell Death and Differentiation*.

[18] Maher P., van Leyen K., Dey P. N., Honrath B., Dolga A., Methner A. (2018). The role of Ca^2+^ in cell death caused by oxidative glutamate toxicity and ferroptosis. *Cell Calcium*.

[19] Wang Z., Guo L. M., Wang Y. (2018). Inhibition of HSP90*α* protects cultured neurons from oxygen-glucose deprivation induced necroptosis by decreasing RIP3 expression. *Journal of Cellular Physiology*.

[20] Xu Y., Ma H. B., Fang Y. L. (2017). Cisplatin-induced necroptosis in TNF*α* dependent and independent pathways. *Cellular Signalling*.

[21] Fearns C., Pan Q., Mathison J. C., Chuang T. H. (2006). Triad3A Regulates Ubiquitination and Proteasomal Degradation of RIP1 following Disruption of Hsp90 Binding. *Journal of Biological Chemistry*.

[22] Hajdinak P., Czobor A., Szarka A. (2019). The potential role of acrolein in plant ferroptosis-like cell death. *PLoS One*.

[23] Sun X. F., Niu X. H., Chen R. C. (2016). Metallothionein-1G facilitates sorafenib resistance through inhibition of ferroptosis. *Hepatology*.

[24] Hanson L. R., Roeytenberg A., Martinez P. M. (2009). Intranasal deferoxamine provides increased brain exposure and significant protection in rat ischemic stroke. *Journal of Pharmacology and Experimental Therapeutics*.

[25] Zacharia G., Shani D., Ortiz R. A. (2017). Recurrent stroke in a patient with vitamin B12deficiency andMTHFRmutation. *Neurology Clinical Practice*.

[26] Guan X. Y., Li X. L., Yang X. J. (2019). The neuroprotective effects of carvacrol on ischemia/reperfusion-induced hippocampal neuronal impairment by ferroptosis mitigation. *Life Sciences*.

[27] Lan B., Ge J. W., Cheng S. W. (2020). Extract of Naotaifang, a compound Chinese herbal medicine, protects neuron ferroptosis induced by acute cerebral ischemia in rats. *Journal of Integrative Medicine*.

[28] Allocca M., Corrigan J. J., Mazumder A., Fake K. R., Samson L. D. (2019). Inflammation, necrosis, and the kinase RIP3 are key mediators of AAG-dependent alkylation-induced retinal degeneration. *Science Signaling*.

[29] Miszczyk J., Rawojć K., Panek A. (2018). Do protons and X-rays induce cell-killing in human peripheral blood lymphocytes by different mechanisms?. *Clinical and Translational Radiation Oncology*.

[30] Zhang S. H., Wang Y. Y., Li D. K., Wu J. F., Wen S., Wu Y. (2016). Necrostatin-1 attenuates inflammatory response and improves cognitive function in chronic ischemic stroke mice. *Medicines*.

[31] Cruz S. A., Qin Z., Stewart A., Chen H. H. (2018). Dabrafenib, an inhibitor of RIP3 kinase-dependent necroptosis, reduces ischemic brain injury. *Neural Regeneration Research*.

[32] Wang J., Liu Y., Li X. H. (2017). Curcumin protects neuronal cells against status-epilepticus-induced hippocampal damage through induction of autophagy and inhibition of necroptosis. *Canadian Journal of Physiology and Pharmacology*.

[33] Sun Y., Zheng Y. F., Wang C. X., Liu Y. Z. (2018). Glutathione depletion induces ferroptosis, autophagy, and premature cell senescence in retinal pigment epithelial cells. *Cell Death & Disease*.

[34] Cao J. Y., Dixon S. J. (2016). Mechanisms of ferroptosis. *Cellular and Molecular Life Sciences*.

[35] Vučković A. M., Bosello Travain V., Bordin L. (2020). Inactivation of the glutathione peroxidase GPx4 by the ferroptosis-inducing molecule RSL3 requires the adaptor protein 14‐3‐3*ε*. *FEBS Letters*.

[36] Forcina G. C., Dixon S. J. (2019). GPX4 at the crossroads of lipid homeostasis and ferroptosis. *Proteomics*.

[37] Dixon S. J., Lemberg K. M., Lamprecht M. R. (2012). Ferroptosis: An iron-dependent form of nonapoptotic cell death. *Cell*.

[38] Badgley M. A., Kremer D. M., Maurer H. C. (2020). Cysteine depletion induces pancreatic tumor ferroptosis in mice. *Science*.

[39] Gao G., Li J., Zhang Y., Chang Y. Z. (2019). Cellular iron metabolism and regulation. *Advances in Experimental Medicine and Biology*.

[40] Seiwert N., Heylmann D., Hasselwander S., Fahrer J. (2020). Mechanism of colorectal carcinogenesis triggered by heme iron from red meat. *Biochimica et Biophysica Acta (BBA) – Reviews on Cancer*.

[41] Ferris C. D., Jaffrey S. R., Sawa A. (1999). Haem oxygenase-1 prevents cell death by regulating cellular iron. *Nature Cell Biology*.

[42] Chifman J., Laubenbacher R., Torti S. V. (2014). A systems biology approach to iron metabolism. *Advances in Experimental Medicine and Biology*.

[43] Oakhill J. S., Marritt S. J., Gareta E. G., Cammack R., Mckie A. T. (2008). Functional characterization of human duodenal cytochrome *b* (Cybrd1): Redox properties in relation to iron and ascorbate metabolism. *Biochimica et Biophysica Acta (BBA) - Bioenergetics*.

[44] Parrow N. L., Li Y., Feola M. (2019). Lobe specificity of iron binding to transferrin modulates murine erythropoiesis and iron homeostasis. *Blood*.

[45] Yan N., Zhang J. J. (2019). Iron metabolism, ferroptosis, and the links with Alzheimer’s disease. *Frontiers in Neuroscience*.

[46] Oosterheert W., van Bezouwen L. S., Rodenburg R. N. P. (2018). Cryo-EM structures of human STEAP4 reveal mechanism of iron(III) reduction. *Nature Communications*.

[47] Zhang F., Tao Y. L., Zhang Z. Z. (2012). Metalloreductase Steap3 coordinates the regulation of iron homeostasis and inflammatory responses. *Haematologica*.

[48] Pattanakuhar S., Phrommintikul A., Tantiworawit A. (2018). Increased sympathovagal imbalance evaluated by heart rate variability is associated with decreased T2∗ MRI and left ventricular function in transfusion-dependent thalassemia patients. *Bioscience Reports*.

[49] Yang L., Wang H., Yang X. (2020). Auranofin mitigates systemic iron overload and induces ferroptosis via distinct mechanisms. *Signal Transduction and Targeted Therapy*.

[50] Ginzburg Y. Z. (2019). Hepcidin-ferroportin axis in health and disease. *Vitamins and Hormones*.

[51] Scindia Y., Leeds J., Swaminathan S. (2019). Iron homeostasis in healthy kidney and its role in acute kidney injury. *Seminars in Nephrology*.

[52] Billesbølle C. B., Azumaya C. M., Kretsch R. C. (2020). Structure of hepcidin-bound ferroportin reveals iron homeostatic mechanisms. *Nature*.

[53] Munoz M., Villar I., Garcia-Erce J. A. (2009). An update on iron physiology. *World Journal of Gastroenterology*.

[54] Yu Y. Y., Jiang L., Wang H. (2020). Hepatic transferrin plays a role in systemic iron homeostasis and liver ferroptosis. *Blood*.

[55] Xin Y. J., Gao H., Wang J. (2017). Manganese transporter Slc39a14 deficiency revealed its key role in maintaining manganese homeostasis in mice. *Cell Discovery*.

[56] Wilkinson N., Pantopoulos K. (2014). The IRP/IRE system in vivo: insights from mouse models. *Frontiers in Pharmacology*.

[57] Lane D., Ayton S., Bush A. I. (2018). Iron and Alzheimer’s disease: an update on emerging mechanisms. *Journal of Alzheimers Disease*.

[58] Park U. J., Lee Y. A., Won S. M. (2011). Blood-derived iron mediates free radical production and neuronal death in the hippocampal CA1 area following transient forebrain ischemia in rat. *Acta Neuropathologica*.

[59] Li G., Li X., Dong J., Han Y. (2021). Electroacupuncture ameliorates cerebral ischemic injury by inhibiting ferroptosis. *Frontier in Neurology*.

[60] Duck K. A., Connor J. R. (2016). Iron uptake and transport across physiological barriers. *Biometals*.

[61] Degregorio-Rocasolano N., Martí-Sistac O., Gasull T. (2019). Deciphering the iron side of stroke: neurodegeneration at the crossroads between iron dyshomeostasis, excitotoxicity, and ferroptosis. *Frontiers in Neuroscience*.

[62] Geburek I., Preiss-Weigert A., Lahrssen-Wiederholt M., Schrenk D., These A. (2020). *In vitro* metabolism of pyrrolizidine alkaloids - Metabolic degradation and GSH conjugate formation of different structure types. *Food and Chemical Toxicology*.

[63] Townsend D. M., Tew K. D., Tapiero H. (2003). The importance of glutathione in human disease. *Biomedicine & Pharmacotherapy*.

[64] Lewerenz J., Hewett S. J., Huang Y. (2013). The cystine/glutamate antiporter system x(c) (-) in health and disease: from molecular mechanisms to novel therapeutic opportunities. *Antioxidants & Redox Signaling*.

[65] Liu J. H., Wang T. W., Lin Y. Y. (2020). Acrolein is involved in ischemic stroke-induced neurotoxicity through spermidine/spermine-N1-acetyltransferase activation. *Experimental Neurology*.

[66] Bersuker K., Hendricks J. M., Li Z. (2019). The CoQ oxidoreductase FSP1 acts parallel to GPX4 to inhibit ferroptosis. *Nature*.

[67] Brütsch S. H., Wang C. C., Li L. (2015). Expression of inactive glutathione peroxidase 4 leads to embryonic lethality, and inactivation of the Alox15 gene does not rescue such knock-in mice. *Antioxidants & Redox Signaling*.

[68] Imai H., Hirao F., Sakamoto T. (2003). Early embryonic lethality caused by targeted disruption of the mouse PHGPx gene. *Biochemical and Biophysical Research Communications*.

[69] Gao M., Yi J., Zhu J. (2019). Role of mitochondria in ferroptosis. *Molecular Cell*.

[70] Jiang X., Andjelkovic A. V., Zhu L. (2018). Blood-brain barrier dysfunction and recovery after ischemic stroke. *Progress in Neurobiology*.

[71] Hambright W. S., Fonseca R. S., Chen L., Na R., Ran Q. (2017). Ablation of ferroptosis regulator glutathione peroxidase 4 in forebrain neurons promotes cognitive impairment and neurodegeneration. *Redox Biology*.

[72] Chu B., Kon N., Chen D. (2019). ALOX12 is required for p53-mediated tumour suppression through a distinct ferroptosis pathway. *Nature Cell Biology*.

[73] Xie Y., Hou W., Song X. (2016). Ferroptosis: process and function. *Cell Death and Differentiation*.

[74] Stockwell B. R., Friedmann Angeli J. P., Bayir H. (2017). Ferroptosis: a regulated cell death nexus linking metabolism, redox biology, and disease. *Cell*.

[75] Ursini F., Maiorino M., Valente M., Ferri L., Gregolin C. (1982). Purification from pig liver of a protein which protects liposomes and biomembranes from peroxidative degradation and exhibits glutathione peroxidase activity on phosphatidylcholine hydroperoxides. *Biochimica et Biophysica Acta (BBA) - Lipids and Lipid Metabolism*.

[76] Kloska A., Malinowska M., Gabig-Cimińska M., Jakóbkiewicz-Banecka J. (2020). Lipids and lipid mediators associated with the risk and pathology of ischemic stroke. *International Journal of Molecular Sciences*.

[77] Xiao F. J., Zhang D., Wu Y. (2019). miRNA-17-92 protects endothelial cells from erastin-induced ferroptosis through targeting the A20-ACSL4 axis. *Biochemical and Biophysical Research Communications*.

[78] Yang W. S., Kim K. J., Gaschler M. M., Patel M., Shchepinov M. S., Stockwell B. R. (2016). Peroxidation of polyunsaturated fatty acids by lipoxygenases drives ferroptosis. *Proceedings of the National Academy of Sciences*.

[79] Wu C., Zhao W., Yu J., Li S., Lin L., Chen X. (2018). Induction of ferroptosis and mitochondrial dysfunction by oxidative stress in PC12 cells. *Scientific Reports*.

[80] Doll S., Proneth B., Tyurina Y. Y. (2017). ACSL4 dictates ferroptosis sensitivity by shaping cellular lipid composition. *Nature Chemical Biology*.

[81] Ayer A., Zarjou A., Agarwal A., Stocker R. (2016). Heme oxygenases in cardiovascular health and disease. *Physiological Reviews*.

[82] Ferretti G., Bacchetti T., Masciangelo S. (2008). Lipid peroxidation in stroke patients. *Clinical Chemistry and Laboratory Medicine*.

[83] Cojocaru I. M., Cojocaru M., Musuroi C., Botezat M., Lazar L., Druta A. (2004). Lipid peroxidation and catalase in diabetes mellitus with and without ischemic stroke. *Romanian Journal of Internal Medicine*.

[84] Gao M., Monian P., Quadri N., Ramasamy R., Jiang X. (2015). Glutaminolysis and transferrin regulate ferroptosis. *Molecular Cell*.

[85] Jennis M., Kung C. P., Basu S. (2016). An African-specific polymorphism in the TP53 gene impairs p53 tumor suppressor function in a mouse model. *Genes & Development*.

[86] Shu R. C., Zhang L. L., Zhang H. (2021). NMDA Receptor Modulates Spinal Iron Accumulation Via Activating DMT1(-)IRE in Remifentanil-Induced Hyperalgesia. *Journal of Pain*.

[87] Krzyżanowska W., Pomierny B., Bystrowska B. (2017). Ceftriaxone- and N-acetylcysteine-induced brain tolerance to ischemia: influence on glutamate levels in focal cerebral ischemia. *PLoS One*.

[88] Datta A., Sarmah D., Mounica L. (2020). Cell death pathways in ischemic stroke and targeted pharmacotherapy. *Translational Stroke Research*.

[89] Sarmah D., Kaur H., Saraf J. (2018). Mitochondrial dysfunction in stroke: implications of stem cell therapy. *Translational Stroke Research*.

[90] Sun M. S., Jin H., Sun X. (2018). Free radical damage in ischemia-reperfusion injury: an obstacle in acute ischemic stroke after revascularization therapy. *Oxidative Medicine and Cellular Longevity*.

[91] Kalogeris T., Baines C. P., Krenz M., Korthuis R. J. (2016). Ischemia/reperfusion. *Comprehensive Physiology*.

[92] Schiffner R., Bischoff S. J., Lehmann T. (2017). Redistribution of cerebral blood flow during severe hypovolemia and reperfusion in a sheep model: critical role of *α*1-Adrenergic signaling. *International Journal of Molecular Sciences*.

[93] Eltzschig H. K., Eckle T. (2011). Ischemia and reperfusion--from mechanism to translation. *Nature Medicine*.

[94] Appel S., Mirakaj V., Bringmann A., Weck M. M., Grunebach F., Brossart P. (2005). PPAR-gamma agonists inhibit toll-like receptor-mediated activation of dendritic cells via the MAP kinase and NF-kappaB pathways. *Blood*.

[95] Fu P., Liu J., Bai Q. (2020). Long-term outcomes of monascin - a novel dual peroxisome proliferator-activated receptor gamma/nuclear factor-erythroid 2 related factor-2 agonist in experimental intracerebral hemorrhage. *Therapeutic Advances in Neurological Disorders*.

[96] Wu X. J., Sun X. H., Wang S. W., Chen J. L., Bi Y. H., Jiang D. X. (2018). Mifepristone alleviates cerebral ischemia-reperfusion injury in rats by stimulating PPAR *γ*. *European Review for Medical and Pharmacological Sciences*.

[97] Wang W., Li X., Ding N. (2020). miR-34a regulates adipogenesis in porcine intramuscular adipocytes by targeting ACSL4. *BMC Genetics*.

[98] Cheng J., Fan Y. Q., Liu B. H., Zhou H., Wang J. M., Chen Q. X. (2020). ACSL4 suppresses glioma cells proliferation via activating ferroptosis. *Oncology Reports*.

[99] Fang S., Yu X., Ding H., Han J., Feng J. (2018). Effects of intracellular iron overload on cell death and identification of potent cell death inhibitors. *Biochemical and Biophysical Research Communications*.

[100] Park E., Chung S. W. (2019). ROS-mediated autophagy increases intracellular iron levels and ferroptosis by ferritin and transferrin receptor regulation. *Cell Death & Disease*.

[101] García-Yébenes I., García-Culebras A., Peña-Martínez C. (2018). Iron overload exacerbates the risk of hemorrhagic transformation after tPA (tissue-type plasminogen activator) administration in thromboembolic stroke mice. *Stroke*.

[102] Fukuta T., Asai T., Yanagida Y. (2017). Combination therapy with liposomal neuroprotectants and tissue plasminogen activator for treatment of ischemic stroke. *FASEB Journal*.

[103] Zeng J., Zhu L., Liu J. (2019). Metformin protects against oxidative stress injury induced by ischemia/reperfusion via regulation of the lncRNA-H19/miR-148a-3p/rock2 axis. *Oxidative Medicine and Cellular Longevity*.

[104] Kaplan P., Tatarkova Z., Sivonova M. K., Racay P., Lehotsky J. (2020). Homocysteine and mitochondria in cardiovascular and cerebrovascular systems. *International Journal of Molecular Sciences*.

[105] Fang X. X., Wang H., Han D. (2019). Ferroptosis as a target for protection against cardiomyopathy. *Proceedings of the National Academy of Sciences of the United States of America*.

[106] Ma S. X., Sun L. Y., Wu W. H., Wu J. L., Sun Z. N., Ren J. J. (2020). USP22 protects against myocardial ischemia-reperfusion injury via the SIRT1-p53/SLC7A11-dependent inhibition of ferroptosis-induced cardiomyocyte death. *Frontiers in Physiology*.

[107] Lillo-Moya J., Rojas-Solé C., Muñoz-Salamanca D., Panieri E., Saso L., Rodrigo R. (2021). Targeting ferroptosis against ischemia/reperfusion cardiac injury. *Antioxidants (Basel)*.

[108] Li N., Jiang W. Y., Wang W., Xiong R., Wu X. J., Geng Q. (2021). Ferroptosis and its emerging roles in cardiovascular diseases. *Pharmacological Research*.

[109] Lu J. J., Xu F., Lu H. (2020). LncRNA PVT1 regulates ferroptosis through miR-214-mediated TFR1 and p53. *Life Sciences*.

[110] Guo H. H., Zhu L. L., Tang P. P. (2021). Carthamin yellow improves cerebral ischemia-reperfusion injury by attenuating inflammation and ferroptosis in rats. *International Journal of Molecular Medicine*.

[111] Ding H., Yan C. Z., Shi H. (2011). Hepcidin is involved in iron regulation in the ischemic brain. *PLoS One*.

[112] Boero M., Pagliaro P., Tullio F. (2015). A comparative study of myocardial molecular phenotypes of two tfr2*β* null mice: role in ischemia/reperfusion. *Biofactors*.

[113] Milanese C., Gabriels S., Barnhoorn S. (2021). Gender biased neuroprotective effect of Transferrin Receptor 2 deletion in multiple models of Parkinson's disease. *Cell Death & Differentiation*.

[114] Shi Z. W., Ge L. S., Li Y. C. (2018). The role of necroptosis in cardiovascular disease. *Frontiers in Pharmacology*.

[115] Liu Y. P., Liu T., Lei T. T. (2019). RIP1/RIP3-regulated necroptosis as a target for multifaceted disease therapy (review). *International Journal of Molecular Medicine*.

[116] Yoon S., Kovalenko A., Bogdanov K., Wallach D. (2017). MLKL, the protein that mediates necroptosis, also regulates endosomal trafficking and extracellular vesicle generation. *Immunity*.

[117] de Almagro M. C., Goncharov T., Izrael-Tomasevic A. (2017). Coordinated ubiquitination and phosphorylation of RIP1 regulates necroptotic cell death. *Cell Death and Differentiation*.

[118] Mompeán M., Li W., Li J. (2018). The structure of the necrosome RIPK1-RIPK3 core, a human hetero-amyloid signaling complex. *Cell*.

[119] Berger S. B., Bertin J., Gough P. J. (2016). Life after death: RIP1 and RIP3 move beyond necroptosis. *Cell Death Discovery*.

[120] Newton K., Dugger D. L., Maltzman A. (2016). RIPK3 deficiency or catalytically inactive RIPK1 provides greater benefit than MLKL deficiency in mouse models of inflammation and tissue injury. *Cell Death and Differentiation*.

[121] Holler N., Zaru R., Micheau O. (2000). Fas triggers an alternative, caspase-8-independent cell death pathway using the kinase RIP as effector molecule. *Nature Immunology*.

[122] Frank D., Vince J. E. (2019). Pyroptosis versus necroptosis: similarities, differences, and crosstalk. *Cell Death and Differentiation*.

[123] Chen J., Kos R., Garssen J., Redegeld F. (2019). Molecular insights into the mechanism of necroptosis: the necrosome as a potential therapeutic target. *Cells*.

[124] Corsetti G., Chen-Scarabelli C., Romano C. (2019). Autophagy and oncosis/necroptosis are enhanced in cardiomyocytes from heart failure patients. *Medical Science Monitor Basic Research*.

[125] Ofengeim D., Yuan J. (2013). Regulation of RIP1 kinase signalling at the crossroads of inflammation and cell death. *Nature Reviews Molecular Cell Biology*.

[126] Huang W., Xie W. D., Gong J. (2020). Heat stress induces RIP1/RIP3-dependent necroptosis through the MAPK, NF-*κ*B, and c-Jun signaling pathways in pulmonary vascular endothelial cells. *Biochemical and Biophysical Research Communications*.

[127] Ea C. K., Deng L., Xia Z. P., Pineda G., Chen Z. J. (2006). Activation of IKK by TNF*α* Requires Site-Specific Ubiquitination of RIP1 and Polyubiquitin Binding by NEMO. *Molecular Cell*.

[128] Moquin D. M., Mcquade T., Chan F. K. (2013). CYLD deubiquitinates RIP1 in the TNF*α*-Induced necrosome to facilitate kinase activation and programmed necrosis. *PLoS One*.

[129] Khoury M. K., Gupta K., Franco S. R., Liu B. (2020). Necroptosis in the pathophysiology of disease. *American Journal of Pathology*.

[130] Ma W. Z., Liu M., Liang F. F. (2020). Cardiotoxicity of sorafenib is mediated through elevation of ROS level and CaMKII activity and dysregulation of calcium homoeostasis. *Basic & Clinical Pharmacology & Toxicology*.

[131] Xiao K., Liu C., Qin Q. (2020). EPA and DHA attenuate deoxynivalenol-induced intestinal porcine epithelial cell injury and protect barrier function integrity by inhibiting necroptosis signaling pathway. *FASEB Journal*.

[132] Zhou H., Li D., Zhu P. (2018). Inhibitory effect of melatonin on necroptosis via repressing the Ripk3-PGAM5-CypD-mPTP pathway attenuates cardiac microvascular ischemia-reperfusion injury. *Journal of Pineal Research*.

[133] Feng S. T., Wang Z. Z., Yuan Y. H. (2020). Dynamin-related protein 1: A protein critical for mitochondrial fission, mitophagy, and neuronal death in Parkinson's disease. *Pharmacological Research*.

[134] He S. D., Wang L., Miao L. (2009). Receptor Interacting Protein Kinase-3 Determines Cellular Necrotic Response to TNF-*α*. *Cell*.

[135] Yuan J. Y., Amin P., Ofengeim D. (2019). Necroptosis and RIPK1-mediated neuroinflammation in CNS diseases. *Nature Reviews Neuroscience*.

[136] Yu X. X., Ruan Y., Huang X. Q. (2020). Dexrazoxane ameliorates doxorubicin-induced cardiotoxicity by inhibiting both apoptosis and necroptosis in cardiomyocytes. *Biochemical and Biophysical Research Communications*.

[137] Gong Y. T., Fan Z. Y., Luo G. P. (2019). The role of necroptosis in cancer biology and therapy. *Molecular Cancer*.

[138] Kearney C. J., Cullen S. P., Tynan G. A. (2015). Necroptosis suppresses inflammation via termination of TNF- or LPS-induced cytokine and chemokine production. *Cell Death and Differentiation*.

[139] Sairanen T., Carpén O., Karjalainen-Lindsberg M. L. (2001). Evolution of cerebral tumor necrosis factor-alpha production during human ischemic stroke. *Stroke*.

[140] Ryan F., Khodagholi F., Dargahi L., Minai-Tehrani D., Ahmadiani A. (2018). Temporal pattern and crosstalk of necroptosis markers with autophagy and apoptosis associated proteins in ischemic hippocampus. *Neurotoxicity Research*.

[141] Tang Y., Le W. (2016). Differential roles of M1 and M2 microglia in neurodegenerative diseases. *Molecular Neurobiology*.

[142] Yang J. P., Zhao Y. Y., Zhang L. (2018). RIPK3/MLKL-mediated neuronal necroptosis modulates the M1/M2 polarization of microglia/macrophages in the ischemic cortex. *Cerebral Cortex*.

[143] Wang H., Liu C., Zhao Y., Gao G. (2020). Mitochondria regulation in ferroptosis. *European Journal of Cell Biology*.

[144] Miyake S., Murai S., Kakuta S., Uchiyama Y., Nakano H. (2020). Identification of the hallmarks of necroptosis and ferroptosis by transmission electron microscopy. *Biochemical and Biophysical Research Communications*.

[145] Xie B. S., Wang Y. Q., Lin Y. (2019). Inhibition of ferroptosis attenuates tissue damage and improves long-term outcomes after traumatic brain injury in mice. *CNS Neuroscience & Therapeutics*.

[146] Ying Y., Padanilam B. J. (2016). Regulation of necrotic cell death: p53, PARP1 and cyclophilin D-overlapping pathways of regulated necrosis?. *Cellular and Molecular Life Sciences*.

[147] Kwong J. Q., Molkentin J. D. (2015). Physiological and pathological roles of the mitochondrial permeability transition pore in the heart. *Cell Metabolism*.

[148] Basit F., van Oppen L. M., Schöckel L. (2017). Mitochondrial complex I inhibition triggers a mitophagy-dependent ROS increase leading to necroptosis and ferroptosis in melanoma cells. *Cell Death & Disease*.

[149] Zhu P. J., Hu S. Y., Jin Q. H. (2018). Ripk3 promotes ER stress-induced necroptosis in cardiac IR injury: a mechanism involving calcium overload/XO/ROS/mPTP pathway. *Redox Biology*.

[150] Matsumoto S., Murozono M., Kanazawa M., Nara T., Ozawa T., Watanabe Y. (2018). Edaravone and cyclosporine A as neuroprotective agents for acute ischemic stroke. *Acute Medicine & Surgery*.

[151] Lamb H. M. (2020). Double agents of cell death: novel emerging functions of apoptotic regulators. *FEBS Journal*.

[152] Karch J., Kanisicak O., Brody M. J., Sargent M. A., Michael D. M., Molkentin J. D. (2015). Necroptosis interfaces with MOMP and the MPTP in mediating cell death. *PLoS One*.

[153] Marshall K. D., Baines C. P. (2014). Necroptosis: is there a role for mitochondria?. *Frontiers in Physiology*.

[154] Zhang T., Zhang Y., Cui M. Y. (2016). CaMKII is a RIP3 substrate mediating ischemia- and oxidative stress-induced myocardial necroptosis. *Nature Medicine*.

[155] Shintoku R., Takigawa Y., Yamada K. (2017). Lipoxygenase-mediated generation of lipid peroxides enhances ferroptosis induced by erastin and RSL3. *Cancer Science*.

[156] Tian Q., Qin B., Gu Y. (2020). ROS-mediated necroptosis is involved in iron overload-induced osteoblastic cell death. *Oxidative Medicine and Cellular Longevity*.

[157] Che L., Yang C. L., Chen Y. (2021). Mitochondrial redox-driven mitofusin 2 *S* -glutathionylation promotes neuronal necroptosis via disrupting ER-mitochondria crosstalk in cadmium- induced neurotoxicity. *Chemosphere*.

[158] Dixon S. J., Patel D. N., Welsch M. (2014). Pharmacological inhibition of cystine-glutamate exchange induces endoplasmic reticulum stress and ferroptosis. *eLife*.

[159] Zhang Y., Su S. S., Zhao S. (2017). RIP1 autophosphorylation is promoted by mitochondrial ROS and is essential for RIP3 recruitment into necrosome. *Nature Communications*.

[160] Lang X., Green M. D., Wang W. (2019). Radiotherapy and immunotherapy promote tumoral lipid oxidation and ferroptosis via synergistic repression of SLC7A11. *Cancer Discovery*.

[161] Abdullah M., Kim D. H., Lee S. J. System x_c_- inhibition shares features of necroptosis and ferroptosis in hepatocellular carcinoma cells.

[162] Li X. G., Cheng S. L., Hu H. (2020). Progranulin protects against cerebral ischemia-reperfusion (I/R) injury by inhibiting necroptosis and oxidative stress. *Biochemical and Biophysical Research Communications*.

[163] Yang C. K., He S. D. (2016). Heat shock protein 90 regulates necroptosis by modulating multiple signaling effectors. *Cell Death & Disease*.

[164] Wu Z. M., Geng Y., Lu X. J. (2019). Chaperone-mediated autophagy is involved in the execution of ferroptosis. *Proceedings of the National Academy of Sciences of the United States of America*.

[165] Zuo Y. C., He T. B., Liao P. Q., Zhuang K., Yan X. X., Liu F. (2020). 17-Allylamino-demethoxygeldanamycin ameliorate microthrombosis via HSP90/RIP3/NLRP3 pathway after subarachnoid hemorrhage in rats. *Acta Neurochirurgica Supplementum*.

[166] Shi W. Z., Tian Y., Li J. (2019). GCN2 suppression attenuates cerebral ischemia in mice by reducing apoptosis and endoplasmic reticulum (ER) stress through the blockage of FoxO3a-regulated ROS production. *Biochemical and Biophysical Research Communications*.

[167] Donze O., Picard D. (1999). Hsp90 binds and regulates the ligand-inducible *α* subunit of eukaryotic translation initiation factor kinase Gcn2. *Molecular and Cellular Biology*.

[168] Chen M. S., Wang S. F., Hsu C. Y. (2017). CHAC1 degradation of glutathione enhances cystine-starvation-induced necroptosis and ferroptosis in human triple negative breast cancer cells via the GCN2-eIF2*α*-ATF4 pathway. *Oncotarget*.

[169] Zhou R., Leng T., Yang T., Chen F., Hu W., Xiong Z. G. (2019). *β*-Estradiol protects against acidosis-mediated and ischemic neuronal injury by promoting ASIC1a (acid-sensing ion channel 1a) protein degradation. *Stroke*.

[170] Zhou R. P., Chen Y., Wei X. (2020). Novel insights into ferroptosis: implications for age-related diseases. *Theranostics*.

[171] Yang X. S., Yi T. L., Zhang S. (2017). Hypoxia-inducible factor-1 alpha is involved in RIP-induced necroptosis caused by *in vitro* and *in vivo* ischemic brain injury. *Scientific Reports*.

[172] Lin C. H., Lee H. T., Lee S. D. (2013). Role of HIF-1*α*-activated Epac1 on HSC-mediated neuroplasticity in stroke model. *Neurobiology of Disease*.

[173] Kim J., Lee H. S., Nam C. M., Heo J. H. (2017). Effects of statin intensity and adherence on the long-term prognosis after acute ischemic stroke. *Stroke*.

[174] Zhao W., Xiao Z. J., Zhao S. P. (2019). The benefits and risks of statin therapy in ischemic stroke: a review of the literature. *Neurology India*.

[175] Zhao J. R., Zhang X. J., Dong L. P., Wen Y., Cui L. L. (2014). The many roles of statins in ischemic stroke. *Current Neuropharmacology*.

[176] Pieters B., Staals J., Knottnerus I. (2009). Periventricular white matter lucencies relate to low vitamin B12 levels in patients with small vessel stroke. *Stroke*.

[177] Yahn G. B., Abato J. E., Jadavji N. M. (2021). Role of vitamin B12 deficiency in ischemic stroke risk and outcome. *Neural Regeneration Research*.

[178] Yang W., Liu X., Song C. (2021). Structure-activity relationship studies of phenothiazine derivatives as a new class of ferroptosis inhibitors together with the therapeutic effect in an ischemic stroke model. *European Journal of Medicinal Chemistry*.

[179] Wang X., Zhang D., Lu X. Y. (2015). Dentate gyrus-CA3 glutamate release/NMDA transmission mediates behavioral despair and antidepressant-like responses to leptin. *Molecular Psychiatry*.

[180] Gao M., Jiang X. (2018). To eat or not to eat -- the metabolic flavor of ferroptosis. *Current Opinion in Cell Biology*.

[181] Busch H. J., Schirmer S. H., Jost M. (2011). Leptin augments cerebral hemodynamic reserve after three-vessel occlusion: distinct effects on cerebrovascular tone and proliferation in a nonlethal model of hypoperfused rat brain. *Journal of Cerebral Blood Flow & Metabolism*.

[182] Zhang W. F., Jin Y. C., Li X. M., Yang Z., Wang D., Cui J. J. (2019). Protective effects of leptin against cerebral ischemia reperfusion injury. *Experimental and Therapeutic Medicine*.

[183] Tao P., Jing Z., Shou-Hong G. (2019). Effects of leptin on norepinephrine in acute ischemic stroke. *Pharmazie*.

[184] Hangel M. S. C. (2019). *Leptin-mediated downregulation of glutathione peroxidase 4 and iron overload contributing to podocyte ferroptosis in diabetic nephropathy*.

[185] Choi Y. H. (2018). Schisandrin A prevents oxidative stress-induced DNA damage and apoptosis by attenuating ROS generation in C2C12 cells. *Biomedicine & Pharmacotherapy*.

[186] Qi J., Liu Y., Yang P. (2015). Heat shock protein 90 inhibition by 17-dimethylaminoethylamino-17-demethoxygeldanamycin protects blood-brain barrier integrity in cerebral ischemic stroke. *American Journal of Translational Research*.

[187] Friedmann Angeli J. P., Schneider M., Proneth B. (2014). Inactivation of the ferroptosis regulator Gpx4 triggers acute renal failure in mice. *Nature Cell Biology*.

[188] Deng X. X., Li S. S., Sun F. Y. (2019). Necrostatin-1 prevents necroptosis in brains after ischemic stroke via inhibition of RIPK1-mediated RIPK3/MLKL signaling. *Aging and Disease*.

[189] Zhou Y., Zhou B., Tu H. (2017). The degradation of mixed lineage kinase domain-like protein promotes neuroprotection after ischemic brain injury. *Oncotarget*.

[190] Zhang Y. Y., Liu W. N., Li Y. Q. (2019). Ligustroflavone reduces necroptosis in rat brain after ischemic stroke through targeting RIPK1/RIPK3/MLKL pathway. *Naunyn-Schmiedeberg's Archives of Pharmacology*.

[191] Abd-Elsameea A. A., Moustaf A. A., Mohamed A. M. (2014). Modulation of the oxidative stress by metformin in the cerebrum of rats exposed to global cerebral ischemia and ischemia/reperfusion. *European Review for Medical and Pharmacological Sciences*.

[192] Guan X., Li Z. H., Zhu S. (2021). Galangin attenuated cerebral ischemia-reperfusion injury by inhibition of ferroptosis through activating the SLC7A11/GPX4 axis in gerbils. *Life Sciences*.

[193] Hardie D. G., Ross F. A., Hawley S. A. (2012). AMPK: a nutrient and energy sensor that maintains energy homeostasis. *Nature Reviews Molecular Cell Biology*.

[194] Wei X., Yi X., Zhu X. H., Jiang D. S. (2020). Posttranslational modifications in ferroptosis. *Oxidative Medicine and Cellular Longevity*.

[195] Lee H., Zandkarimi F., Zhang Y. (2020). Energy-stress-mediated AMPK activation inhibits ferroptosis. *Nature Cell Biology*.

[196] Liu T., Song F., Lu D. Y. (2018). Anti-oxidation and anti-apoptosis mechanism of Xinshao formula on cerebral ischemia reperfusion injury. *Zhongguo Zhong Yao Za Zhi*.

[197] Cao J. Y., Chen Z. Y., Zhu Y. R. (2014). Huangqi−Honghua combination and its main components ameliorate cerebral infarction with Qi deficiency and blood stasis syndrome by antioxidant action in rats. *Journal of Ethnopharmacology*.

[198] Zhang P., Li W., Li L. (2012). Treatment with edaravone attenuates ischemic brain injury and inhibits neurogenesis in the subventricular zone of adult rats after focal cerebral ischemia and reperfusion injury. *Neuroscience*.

[199] Fakharnia F., Khodagholi F., Dargahi L., Ahmadiani A. (2017). Prevention of cyclophilin D-mediated mPTP opening using cyclosporine-A alleviates the elevation of necroptosis, autophagy and apoptosis-related markers following global cerebral ischemia-reperfusion. *Journal of Molecular Neuroscience*.

[200] Yang R. L., Hu K., Chen J. Y. (2017). Necrostatin-1 protects hippocampal neurons against ischemia/reperfusion injury via the RIP3/DAXX signaling pathway in rats. *Neuroscience Letters*.

[201] Liu J., Zhu Y. M., Guo Y. (2020). Inhibition of GSK3*β* and RIP1K attenuates glial scar formation induced by ischemic stroke via reduction of inflammatory cytokine production. *Frontiers in Pharmacology*.

[202] Yang M., Lv Y. J., Tian X. C. (2017). Neuroprotective effect of *β*-Caryophyllene on cerebral ischemia-reperfusion injury via regulation of necroptotic neuronal death and inflammation: in vivo and in vitro. *Frontiers in Neuroscience*.

[203] Tian J., Guo S., Chen H. (2018). Combination of emricasan with ponatinib synergistically reduces ischemia/reperfusion injury in rat brain through simultaneous prevention of apoptosis and necroptosis. *Translational Stroke Research*.

[204] Xian W. J., Li T., Li L. Y., Hu L. S., Cao J. (2019). Maresin 1 attenuates the inflammatory response and mitochondrial damage in mice with cerebral ischemia/reperfusion in a SIRT1-dependent manner. *Brain Research*.

[205] Khoshnam S. E., Sarkaki A., Rashno M., Farbood Y. (2018). Memory deficits and hippocampal inflammation in cerebral hypoperfusion and reperfusion in male rats: neuroprotective role of vanillic acid. *Life Sciences*.

[206] Huang D., Yang C. Z., Wang Y., Liao Y. H., Huang K. (2009). PARP-1 suppresses adiponectin expression through poly (ADP-ribosyl) ation of PPAR gamma in cardiac fibroblasts. *Cardiovascular Research*.

[207] Lu L. Q., Tian J., Luo X. J., Peng J. (2021). Targeting the pathways of regulated necrosis: a potential strategy for alleviation of cardio-cerebrovascular injury. *Cellular and Molecular Life Sciences*.

